# Recommendations for updating regulations on advertising, promotion and sponsorship of tobacco and nicotine products in the European Union

**DOI:** 10.18332/tpc/204275

**Published:** 2025-05-29

**Authors:** Helena Koprivnikar, Dolors Carnicer-Pont, Anna M. López, Adrián González-Marrón, Gunnar Sæbø, Silvano Gallus, Irene Possenti, Angeliki Lambrou, Efstathios Papachristou, Melinda Pénzes, Sotiria Schoretsaniti, Naia Arteta, Esteve Fernández

**Affiliations:** 1 National Institute of Public Health, Ljubljana, Slovenia; 2 Institut Catala d’Oncologia, ICO, Hospitalet de Llobregat, Barcelona, Spain; 3 Bellvitge Biomedical Research Institute, IDIBELL, Hospitalet de Llobregat, Barcelona, Spain; 4 CIBER in Respiratory Diseases, CIBERES, Madrid, Spain; 5 Group of Evaluation of Health Determinants and Health Policies, Department of Basic Sciences, Universitat Internacional de Catalunya, Sant Cugat del Valles, Spain; 6 Νorwegian Institute of Public Health, Oslo, Norway; 7 Department of Medical Epidemiology, Istituto di Ricerche Farmacologiche Mario Negri IRCCS, Milan, Italy; 8 Directorate of Epidemiology and Prevention of Non-Communicable Diseases & Injuries, National Public Health Organization (NPHO), Athens, Greece; 9 Health Services Management Training Centre, Semmelweis University, Budapest, Hungary; 10 National Koranyi Institute of Pulmonology, Budapest, Hungary; 11 Sociedad Vasconavarra para la Prevencion del Tabaquismo, Bilbao, Spain; 12 Department of Clinical Sciences, School of Medicine and Health Sciences, Universitat de Barcelona, Spain

**Keywords:** tobacco, nicotine, legislation, advertising, law enforcement

## Abstract

**INTRODUCTION:**

Comprehensive bans on advertising, promotion and sponsorship of tobacco and nicotine products (TAPS) have proven effective in reducing their use. The Joint Action on Tobacco Control 2 (JATC2) aims to identify TAPS gaps in the current European Union (EU) regulations and to provide comprehensive recommendations for updating them.

**METHODS:**

An online consultation with European TAPS national experts was conducted in 2023. Seventy-seven experts from 27 European countries were contacted and 38 experts from 21 countries participated in the consultation.

**RESULTS:**

Significant gaps in current TAPS regulations were identified, particularly in entertainment, online media and points of sale. Citizens are not adequately protected from TAPS, the tobacco industry extensively uses loopholes to circumvent regulations. TAPS-related issues currently affect tobacco and particularly non-therapeutic nicotine products, devices, accessories, products imitations and all marketing channels, entertainment, online media and especially, social networks. To address these challenges, regulations should include bans on internet sales and TAPS at points of sale, licensing, decreased retail availability, plain packaging and ban on corporate social responsibility actions, corporate promotion and ‘brand stretching’. These measures should be accompanied by effective monitoring and enforcement, dissuasive sanctions, formalized collaboration among countries and international collaboration, civil society involvement, strong public education, and community awareness programs.

**CONCLUSIONS:**

There is an urgent need to address the current gaps in the EU TAPS regulations through comprehensive and harmonized TAPS bans across all EU countries. Updated regulations must anticipate emerging industry strategies and new products, ensuring continuous adaptation to counteract them effectively.

## INTRODUCTION

The tobacco epidemic is one of the leading preventable public health problems in Europe^1^. Currently, the most significant obstacles to reducing this epidemic include advertising, promotion, and sponsorship of tobacco and nicotine products [combustible tobacco products, smokeless tobacco products, heated tobacco products (HTPs), electronic cigarettes (e-cigarettes), nicotine pouches, heated herbal products with nicotine, etc.], hereinafter referred to as TAPS^2,3^. TAPS increase smoking initiation^3-5^, progression of tobacco use among young people^4^, and tobacco use in the general population^3,4^. Young people are the key target group of TAPS^4^. There is scant research regarding the effects of TAPS and TAPS bans on the perceptions, intentions to use, and consumption of new tobacco and nicotine products. However, for e-cigarettes, the available research shows associations of exposure with lower harm perceptions, increased intention to use, and trial of e-cigarettes^6,7^. Despite some evidence suggesting that nicotine-containing e-cigarettes could aid smoking cessation^8^, minimal research exists on the effects of advertising e-cigarettes for smoking cessation. Given their vulnerability, adolescents should be protected from TAPS^9^.

The World Health Organization (WHO) Framework Convention on Tobacco Control (FCTC) encourages countries to implement a comprehensive TAPS ban and adopt measures that extend beyond the obligations listed in the Convention and its protocols^3,9^. Comprehensive bans reduce exposure to TAPS^10^ and reduce tobacco consumption^3,4,10^, whereas partial bans have minimal effects^4^.

In the European Union (EU), TAPS is currently regulated by the Tobacco Advertising Directive 2003/33/EC^11^, the Tobacco Products Directive 2014/40/EU^12^ and the Audio-visual Media Services Directive 2010/13/EU, amended in 2018^13^. The Tobacco Advertising Directive bans cross-border advertising and sponsorship in print media, radio, and the internet and sponsorship of events involving several EU Member States (MS) as well as having cross border effects, including a ban on free distribution of products^11^. The Tobacco Products Directive defines the same bans for e-cigarettes and refill containers^12^. The Audio-visual Media Services Directive Ban focuses on audio-visual commercial communications, sponsorship, and product placement for tobacco products, e-cigarettes, and refill containers in all forms of audio-visual commercial communications^13^. Despite these regulations, significant gaps remain in both EU and national TAPS regulations and their enforcement. The 2023 Eurobarometer researching attitudes regarding points of sale and plain packaging for cigarettes, e-cigarettes, and HTPs reveals that public opinion is in favor of keeping all products out of sight at points of sale and introducing plain packaging. Furthermore, countering tobacco industry tactics is highlighted as a crucial element by the WHO report on the global tobacco epidemic^2^.

Within the Work Package 8 (WP8) of the Joint Action on Tobacco Control 2 (JATC2), an online consultation was conducted in May and June 2023 with European TAPS national experts from the fields of regulation, research, enforcement, and non-governmental organizations^14^.

This article aims to formulate recommendations for updating EU TAPS regulations based on identified gaps in TAPS regulations, through expert consultation and EU reports.

## METHODS

### Study design

This is a descriptive analysis of the data (quantitative and qualitative) obtained through expert consultations. The online questionnaire contained two sections: Section 1, with 25 questions on recent TAPS regulation changes at the national level, current TAPS problems (domestic and cross-border), the main reasons behind TAPS problems, and suggestions to improve current regulation on TAPS; and Section 2 on identified best practices on TAPS within the experts’ country.

Experts were retrieved from the list of stakeholders identified in the JATC2 and other sources, such as networks and associations devoted to tobacco control research and advocacy in Europe. The criterion for selecting experts was their involvement in any area of tobacco and/or nicotine control.

### Variables

Questions about changes in TAPS regulation over the last three years, current problems, and measures taken to overcome TAPS issues were either free-response or closed-ended (e.g. multiple choice, Likert scale response options).

The current problems were rated according to the perceived importance (high, moderate, low, none, don’t know) by the respondent for each one of the following seven types of tobacco-related products: tobacco products for smoking (cigarettes, roll-your-own, cigars, cigarillos, pipes, waterpipes); heated tobacco products sticks; heated tobacco products devices; tobacco for oral use, snuffing or chewing; e-cigarette liquids; e-cigarette devices; and new products containing nicotine such as nicotine pouches.

The main reasons behind TAPS problems for each of the above-mentioned tobacco-related products were classified into six multiple-choice options: 1) gaps in current regulation, 2) problems with the implementation of regulation, 3) problems in monitoring and enforcement, 4) low compliance with regulation, 5) new approaches by the industry, and 6) don’t know.

The respondents were asked to rate the perceived importance of the predefined list of seven main gaps in TAPS regulation: gaps in definitions of rules and regulations, gaps regarding events with a cross-border dimension, exclusion of devices from TAPS bans, gaps concerning novel products, the possibility of cross-border internet sales, gaps in internet advertising, and gaps in social media.

Additionally, a list of eight suggestions to improve current TAPS regulation was asked to be rated: 1) Provisions on TAPS in EU regulation covering all emerging products, including heated tobacco products and their devices; 2) Social media advertising more clearly included and covered by EU regulation; 3) A broader definition of advertising, which includes the behavior of smoking (to prevent visuals of people smoking in social media posts, articles, or apps); 4) TAPS bans accompanied by an efficient enforcement mechanism; 5) EU-level online compliance tool; 6) Mandatory reporting of tobacco industry promotional expenditures; 7) Greater cooperation between MS to improve the enforcement system (including the exchange of best practices, discussion on challenges faced, and steps taken to overcome them); and 8) Collaboration between MS and other relevant stakeholders (for example, civil society organizations, global initiatives, citizens and audio-visual services regulators).

To explore current problems the questionnaire related them within a list of 24 different TAPS areas grouped into six TAPS themes in line with the 2021 EU report^15^ (Supplementary file Table 1). We performed content analysis by categorizing the word terms and meanings of the responses of experts addressing gaps and proposing solutions to improve TAPS regulation to match the above grouping of six TAPS themes.

## RESULTS

After contacting 77 experts from 26 European countries (Austria, Belgium, Bulgaria, Czechia, Cyprus, Denmark, Estonia, Finland, France, Germany, Greece, Hungary, Ireland, Italy, Latvia, Lithuania, Luxembourg, Malta, Netherlands, Poland, Portugal, Romania, Slovenia, Spain, Sweden), the United Kingdom and Norway, 38 experts from 21 EU MS participated in the consultation, with each country contributing one to four respondents. Most respondents (66%) came from governmental bodies, such as the Department of Health, Ministry of Health, and Health Service Executive, reflecting the involvement of authoritative bodies in the survey. NGOs, universities, and other institutions accounted for 18%, 8%, and 5%, respectively. Positions held by participants varied widely (Supplementary file Table 2).

Respondents from five countries reported changes in TAPS regulation between 2021 and 2023: extension of the display ban to HTPs’ devices; prohibition of drawings (e.g. lottery prizes) for tobacco products and e-cigarettes; restrictions on visibility of tobacco products in shops; extension of TAPS regulation to tobacconist stores; inclusion of nicotine products (e.g. nicotine pouches) in regulation.

### Extent and reasons behind TAPS problems by type of tobacco or nicotine product

There were 20 out of 38 (53%) respondents who referred to the extent of current TAPS problems. Those rated as high extent were mainly related to e-cigarette devices (10 out of 20; 50%) and new products containing nicotine, such as nicotine pouches (9 out of 20; 45%). TAPS problems identified as being of moderate extent were mainly related to e-cigarette liquids and devices (Table 1).

**Table 1:** TAPS problems and reasons behind them by type of tobacco/nicotine product

	*Tobacco products for smoking*	*Tobacco for oral use, snuffing or chewing*	*HTP sticks*	*HTPs devices*	*E-cigarette liquids*	*E-cigarette devices*	*New products with nicotine*
	*n (%)*	*n (%)*	*n (%)*	*n (%)*	*n (%)*	*n (%)*	*n (%)*
**Extent of the TAPS issue**							
High	2 (10)	4 (20)	3 (15)	7 (35)	6 (30)	10 (50)	9 (45)
Moderate	7 (35)	4 (20)	3 (15)	2 (10)	13 (65)	9 (45)	4 (20)
Low/none/don’t know	11 (55)	12 (60)	14 (70)	11 (55)	1 (5)	1 (5)	7 (35)
Total	20 (100)	20 (100)	20 (100)	20 (100)	20 (100)	20 (100)	20 (100)
**Reasons behind TAPS issue**							
Emergence of new approaches by the industry	5 (29)	7 (44)	3 (38)	4 (29)	8 (30)	10 (31)	6 (29)
Gaps in current regulation	4 (24)	4 (25)	1 (13)	4 (29)	10 (37)	10 (31)	9 (43)
Problems with monitoring and enforcement	6 (35)	6 (38)	3 (38)	4 (29)	6 (22)	10 (31)	4 (19)
Low compliance with the regulation	4 (24)	3 (19)	2 (25)	3 (21)	8 (30)	8 (25)	4 (19)
Problems with implementation of regulation	3 (18)	3 (19)	2 (25)	3 (21)	3 (11)	4 (13)	4 (19)
Total	17 (100)	16 (100)	8 (100)	14 (100)	27 (100)	32 (100)	21 (100)

Among the 24 TAPS areas rated by perceived extent of the TAPS problem, the most frequently rated as high or moderate extent were non-retailer websites (e.g. search engines, news services), social media, app stores (8 out of 38; 21%); national or local TV adverts (7 out of 38; 18%) and corporate social responsibility (5 out of 38; 13%) (Supplementary file Table 3).

The question regarding the reasons behind TAPS problems was answered only by those who rated the problems as high or moderate in the previous question. The most frequently mentioned reasons behind TAPS problems, considering the type of tobacco and nicotine product, were: the emergence of new industry approaches (43 mentions); gaps in existing regulations (42 mentions); difficulties in monitoring and enforcement (39 mentions); low compliance rates (32 mentions); and challenges with regulatory implementation (22 mentions) (Table 1).

### Gaps in the current TAPS regulation

Among the list of seven predefined gaps in the current TAPS regulation, the 38 experts most frequently identified the following as high or moderate importance: ‘Gaps on social media’ and ‘Gaps on internet advertisements’ (35 out of 38; 92%); ‘The possibility of cross-border internet sales’ and ‘Gaps concerning novel products’ (28 out of 38; 74%); ‘Exclusion of devices from TAPS bans’ (23 out of 38; 60%); ‘Gaps regarding events with cross-border dimension’ and ‘Gaps in definitions of rules and regulations’ (17 out of 30; 45%) (Table 2).

**Table 2:** TAPS gaps and proposed solutions according to their perceived importance by the respondents (N=38)

	*High*	*Moderate*	*Low, none, don’t know*
	*n (%)*	*n (%)*	*n (%)*
**Gaps in current TAPS regulations**			
Gaps on social media	28 (74)	7 (18)	3 (8)
Gaps on internet advertisements	28 (74)	7 (18)	3 (8)
The possibility of cross-border internet sales	20 (53)	8 (21)	10 (26)
Gaps concerning novel products	20 (53)	8 (21)	10 (26)
Exclusion of devices from TAPS bans	13 (34)	10 (26)	15 (39)
Gaps regarding events with cross-border dimension	10 (26)	7 (18)	21 (55)
Gaps in definitions of rules and regulations	6 (16)	11 (29)	21 (55)
**Suggestions to improve current TAPS regulation**			
Social media advertising more clearly include and covered by EU regulation	31 (82)	6 (16)	1 (3)
Greater cooperation between MS to improve enforcement system	21 (55)	14 (37)	3 (8)
Broader definition of advertising which includes the behavior smoking	25 (66)	9 (24)	4 (11)
Provision on TAPS in EU regulation covering all emerging products	26 (68)	7 (18)	5 (13)
Collaboration between MS and other stakeholders	15 (39)	18 (47)	5 (13)
TAPS bans accompanied by an efficient enforcement mechanism	26 (68)	6 (16)	6 (16)
Mandatory reporting of tobacco industry promotional expenditures	18 (47)	11 (29)	9 (24)
EU-level online compliance tool	18 (47)	11 (29)	9 (24)

More specifically, in open responses, the experts highlighted the following:

1. General gaps in TAPS: lack of updating the TAPS regulations; substantial gaps in the regulation of new products; challenges defining and regulating sponsorship, especially indirect sponsorship activities^14,15^; lack of specific definitions (commercial communication, specialist shops)^14-17^.
2. Gaps related to billboards, posters, and other types of advertising outside the home: advertising in public spaces, newspapers or magazines, railway stations, airports, inflight magazines, and national or local TV advertising^14,15,17^.
3. Gaps related to points of sale, sample, giveaways, promotional items and direct marketing: in the points of sales, there are products visible on display, promotional items, sales promotion and free trial of products^14,15^; duty-free sales are often exceptions to TAPS bans^14^; and promotion campaigns under the guise of corporate social responsibilities^14,15^.
4. Gaps related to internet, social media and mobile applications: there are many products’ depictions in entertainment media content, TV shows, films, online social networks or blogs, non-retailer websites and streaming services; there is widespread online TAPS (direct or indirect), especially for new tobacco and nicotine products^14-17^; with widespread influencer marketing on social media, many posts look like festivals and summer scenes^14,17^; not all social media fall under the Audio-visual Media Services Directive, generating different approaches to TAPS depending on the social media; the scope of regulation of social media remains unclear (private groups, accounts linked to the tobacco industry with posted promotional materials, friend-to-friend advertising, etc.)^14,15,17^; and an unclear distinction exists between paid advertisements and posts by consumers or influencers^14,15^.
5. Gaps related to sponsorship, corporate responsibility, corporate promotion, and other public relation tactics, ‘brand stretching’ and imitation products: sponsorship of events in countries outside the EU but broadcast in the EU; and direct or indirect TAPS at sports events, cultural events, also with free distribution of products^14,15,17^.
6. Gaps related to the monitoring and enforcement of TAPS bans: lack of financial and human resources^14,15^; administrative burdens or delays in addressing violations; and high litigation costs, dealing with internationally operating companies with their own legal departments^14,15,17^ (Supplementary file Table 4).

### Proposed solutions to address the gaps in the current TAPS regulation

Among the list of eight suggestions in the questionnaire to improve current TAPS regulation, the 38 experts most frequently rated the following three as being of high or moderate importance: ‘Social media advertising to be included and more clearly covered by EU regulation’ (37 out of 38; 97%); ‘Greater cooperation between Member States to improve the enforcement system (including exchange of best practices, discussion on challenges faced and steps taken to overcome them)’ (35 out of 38; 92%); ‘A broader definition of advertising, which includes the behavior of smoking (to prevent visuals of people smoking in social media posts, articles, or apps)’ (34 out of 38; 89%) (Table 2).

Within open responses, national experts proposed the following solutions for current gaps in TAPS regulation:

1. General measures: clear, comprehensive legislation closing the current loopholes and keeping pace with rapidly evolving products and TAPS methods^14,15^; comprehensive EU level regulations harmonizing and strengthening existing laws^14,17^; regulations covering all emerging products and devices, as well as channels^14,15^; a broader definition of smoking, including smoking behavior^14,15,17^; mandatory reporting of the tobacco industry’s promotional expenditures^14,17^; plain packaging^14^; and raising public awareness on TAPS^14^.
2. Related to points of sale, samples, giveaways, promotional items, and direct marketing: display bans^14^.
3. Related to internet, social media, and mobile applications: regulations covering social media advertising more clearly; specific and stricter provisions regarding social media^14,15,17^; guidance for and cooperation with social media and regulation of influencers regarding TAPS^14^; and bans on online sales^14^.
4. Related to sponsorship, corporate responsibility, corporate promotion, and other public relation tactics, ‘brand stretching’ and imitation products: comprehensive ban on corporate social responsibility activities and corporate promotion^14^; and a comprehensive ban on the production and distribution of items such as sweets, snacks, and toys, or other products that resemble cigarettes or products, devices, and accessories^14^.
5. Related to monitoring and enforcement: improved efficiency of monitoring and enforcement systems, adequate resource allocation (human, financial, technical), reduced administrative burdens, increased enforcement power and administrative decisions, and sanctions^14,15,17^; EU level compliance tool^14,15,17^; increased cooperation among the EU Member States and other international exchange of best practices, EU coordination^14,15,17^; cooperation with other relevant stakeholders (civil society organizations, citizens, NGOs, audio-visual services regulators)^14,17^; and notifying the industry and other relevant entities about the regulations^14^ (Supplementary file Table 5).

## DISCUSSION

To be effective, TAPS bans must be comprehensive, meaning they completely cover all forms of TAPS activities in all types of media for all types of tobacco and nicotine products^9^. Expert consultation revealed multiple gaps in TAPS regulation across the EU^14-16^. Only four EU MS and Norway reported changes in TAPS regulations between 2021 and 2023^14^, few intend to extend it^15^, and few are striving to ensure that new nicotine products are regulated in a similar way to tobacco^14^. Therefore, the following recommendations on TAPS bans in the EU should be considered.

### Comprehensive ban, harmonized to all EU countries

This should apply to all forms of communication, action and contribution, emerging marketing channels, existing and new tobacco and nicotine products, devices and accessories for use, product imitations and all individuals and entities involved in TAPS. A ban on TAPS is effective only if it has a broad scope, otherwise the tobacco industry inevitably shifts to other strategies^5^. Also, TAPS regulations must be appropriately designed for future industry strategies and products, and must continuously progress, as they can be quickly outdated^11^.

### Comprehensive ban on billboards, posters, and other types of advertising outside the home

This should be applied especially for new nicotine products which are not yet regulated in the EU^15,16^.

### Comprehensive ban at points of sale and decrease in retail availability

This should be applied since tobacco retailers are a key pillar in TAPS^18^ with the effect of promoting tobacco and nicotine product use, especially among youth^6,27^. Display of tobacco products and vending machines at points of sale are a type of TAPS^8^. Moreover, advertising and promotion of smoking accessories, such as cigarette papers, filters, equipment for rolling cigarettes and imitations of tobacco products, among others, may also promote the consumption of these products^9^. In countries with TAPS bans at points of sale, the prevalence of smoking is significantly lower among adolescents, suggesting that these bans can reduce youth smoking^28^. The ban should also apply to ferries, airplanes, ports and airports. Only the textual listing of products and their prices, without any promotional elements, should be allowed^9^. The majority of the EU MS ban advertising^17^ and promotional activities at points of sales for traditional tobacco products, e-cigarettes and HTPs^17^; eight EU MS ban display^16^ and six ban vending machines for these products^29^. The tobacco industry offers retailers agreements with incentives^30^ that enable substantial manufacturer control of tobacco product availability, placement, pricing and promotion in the retail setting^30^. Promotional activities to retailers should be banned^30^. The widespread retail availability of tobacco weakens actions to reduce TAPS connected to retail^18^. Therefore, retail availability should be reduced. Tobacco sale at educational establishments, hospitality, sporting, healthcare centers, entertainment, music, dance and social venues or events should be banned. Licensing of manufacturers, wholesalers and retailers is another effective method for controlling TAPS^9,10^.

### Comprehensive TAPS ban in entertainment, internet, social media and mobile applications

This is the major gap in the EU TAPS regulations^14,15^, has vast reach to youth^19^ and is highly effective because it is ubiquitous, interactive and personalized^20^. In countries with TAPS bans, up to one-third of adolescents still report exposure to TAPS online, which implies that current regulations do not adequately protect youth^21^. The depiction of tobacco in entertainment media products can strongly influence tobacco use, particularly among youth^3,9,19^, even if it is brief ^3,4,10^. Digital content is cross-border in nature, and service providers can be located in a different country than the one where the service is accessed^19^. Thus, TAPS in digital media are challenging to regulate^14^, especially when content is produced abroad^16^. Cross-border digital media use provides emerging channels for TAPS that are outside of existing regulatory control for governments^20^. Next-generation digital technologies (virtual reality, augmented reality, blockchain technology) and the growth of new digital spaces (metaverse), will very likely offer additional opportunities for TAPS, which might occur even further out of regulators’ eyesight and in spaces where jurisdiction is unclear, posing additional challenges to regulation^20^. Each social media platform sets its own restrictions on TAPS^22^, but self-imposed policies of social media platforms are insufficient to restrict TAPS^18,22^, therefore, it is imperative to regulate TAPS and include age verification in social media platforms at the EU level^22^. Digital media communication platforms must apply and enforce existing TAPS bans in accordance with the national laws^19^. Moreover, media productions with smoking imagery should be ineligible for public subsidies^23^. There should be an intensive use of new technologies to assist in eliminating cross-border TAPS^23^, such as the Tobacco Enforcement and Reporting Movement, which is an artificial intelligence-powered digital media monitoring system^20^.

### Comprehensive TAPS ban on internet sales

This should apply not only to not only to entities that sell the products^9,25^ but also to all individuals and entities engaged in any transaction^25^. Through online sales, products such as snus are available to customers in the EU MS, where the sale of these products is banned, and so the industry contravenes regulations^26^. Internet sales also pose additional problems, such as sales to minors and challenging monitoring and enforcement, especially when domains are registered in another country^14,15^. Currently, at least 16 EU MS have bans on online sales of traditional tobacco products for smoking, e-cigarettes and HTPs by specialist retailers^15^.

### Comprehensive ban on corporate social responsibility actions, corporate promotion and ‘brand stretching’

This should be applied, although most EU MS have already implemented such bans^15^. Some countries report that it is difficult to determine in court when such actions represent TAPS^16^. Corporate social responsibility actions and corporate promotion enable access to and dialogue with policymakers for the tobacco industry in order to influence policymaking, which underlines the need for stronger implementation of Article 5.3 of the WHO FCTC^35^. ‘Brand stretching’ (also known as ‘brand extension’ or ‘brand sharing’) is a marketing strategy where a company uses its existing brand name on a new product and should be regarded as a sponsorship form of TAPS^9^. Just over a half of EU MS have implemented total bans on ‘brand stretching’ and imitation products for traditional tobacco products for smoking, e-cigarettes and HTPs^15^. Also, the comprehensive TAPS ban on the production and distribution of items such as sweets, snacks and toys or other products that resemble cigarettes or products, devices and accessories may help achieve public health goals of reducing tobacco use in youth as these products promote consumption^9,35,36^. At least six EU MS have already introduced these bans; half of them ban manufacturing and selling sweets, snacks, toys, or other items resembling tobacco products intended for people under the age of 18 years^29^.

### Strong enforcement and monitoring of the TAPS ban with the EU wide and international collaboration, implementation of public education and community awareness program

This is the most efficient way to eliminate cross-border TAPS^19^. Most EU MS have provisions in place to ensure monitoring and enforcement of national TAPS regulations. Challenges are mainly the lack of human resources, administrative burdens or delays, and instances in cross-border TAPS in which it is difficult to conduct inspections and determine responsibilities^15^. Enforcement of TAPS bans can be improved by having an EU harmonized enforcement system and compliance tool, especially for regulating digital entertainment media^19^. Given the covert nature of TAPS and the difficulty of identifying and reaching offenders, special domestic resources are needed to make enforcement and monitoring of these bans operational^9^. The tobacco industry should be required to disclose all expenditures associated with TAPS^19^. Wide definition of responsible entities, dissuasive penalties, civil society and citizens’ involvement in the monitoring and effective enforcement of the ban, are necessary^9^. Public awareness of the need to eliminate TAPS should be promoted and strengthened in all sectors of the society^9^.

### Plain packaging

Packaging is an important element of TAPS, decreased by plain packaging, which should apply to all different tobacco and nicotine products^31,32^.

### Limitations

We derive some recommendations based on different sources of information that are, in turn, subject to some potential sources of error. First, the consultation with experts was a cross-sectional qualitative survey. We cannot disregard participation bias due to the strategy of recruiting volunteer participants based on the stakeholders identified within the JATC2 and other European networks. No census of experts exists, preventing any kind of sampling, and participation was voluntary, but the response rate was almost 50%. We succeeded in recruiting experts not affiliated with government bodies (e.g. national or regional Ministries of Health or Public Health Agencies), thus minimizing potential self-complacency bias. The combination of these sources of information provides a reliable picture of the gaps and solutions to improve TAPS regulations in the EU and its MS. The way in which our recommendations may have implications beyond the EU borders is out of the scope of this work.

## CONCLUSIONS

There is an urgent need to address the current gaps in the EU TAPS regulations and ensure collaboration among the EU MS to counteract both domestic and cross-border TAPS activity. The EU and its MS should implement, in a harmonized way, comprehensive TAPS bans affecting all products, devices, accessories, product imitations, and marketing channels to prevent the industry from shifting to unattended ways to promote tobacco products and tobacco use. Strong coordinated monitoring and enforcement of the legislation should be ensured, involving civil society, dissuasive penalties for widely defined responsible entities and new technical solutions for monitoring.

## Supplementary Material



## Data Availability

The data supporting this research are available from the authors on reasonable request.

## References

[1] World Health Organization. The European Health Report 2021: Taking stock of the health-related Sustainable Development Goals in the COVID-19 era with a focus on leaving no one behind. WHO; 2022. Accessed April 24, 2024. https://iris.who.int/bitstream/handle/10665/352137/9789289057547-eng.pdf?sequence=2

[2] World Health Organization. WHO global report on trends in prevalence of tobacco use 2000-2030. WHO; 2024. Accessed April 24, 2024. https://iris.who.int/bitstream/handle/10665/375711/9789240088283-eng.pdf?sequence=1

[3] WHO Framework Convention on Tobacco Control. Report of the Expert Group on Tobacco Advertising, Promotion and Sponsorship: Depiction of Tobacco in Entertainment Media. WHO; 2018. Accessed April 24, 2024. https://iris.who.int/bitstream/handle/10665/371014/fctc-cop8-supp-inf-2-en.pdf?sequence=1&isAllowed=y

[4] National Cancer Institute. The Role of the Media in Promoting and Reducing Tobacco Use. Tobacco Control Monograph No. 19. U.S. Department of Health and Human Services, National Institutes of Health, National Cancer Institute; 2008. NIH Pub. No. 07-6242, June 2008. Accessed April 24, 2024. https://cancercontrol.cancer.gov/sites/default/files/2020-08/m19_complete.pdf

[5] Lovato C, Watts A, Stead LF. Impact of tobacco advertising and promotion on increasing adolescent smoking behaviours. Cochrane Database Syst Rev. 2011;2011(10):CD003439. doi:10.1002/14651858.CD003439.pub2 21975739 PMC7173757

[6] Cruz TB, McConnell R, Low BW, et al. Tobacco Marketing and Subsequent Use of Cigarettes, E-Cigarettes, and Hookah in Adolescents. Nicotine Tob Res. 2019;21:926-932. doi:10.1093/ntr/nty107 29846704 PMC6588392

[7] Collins L, Glasser AM, Abudayyeh H, et al. E-Cigarette Marketing and Communication: How E-Cigarette Companies Market E-Cigarettes and the Public Engages with E-cigarette Information. Nicotine Tob Res. 2019;21(1):14-24. doi:10.1093/ntr/ntx284 29315420 PMC6610165

[8] Lindson N, Butler AR, McRobbie H, et al. Electronic cigarettes for smoking cessation. Cochrane Database Syst Rev. 2024;1(1):CD010216. doi:10.1002/14651858.CD010216.pub8 38189560 PMC10772980

[9] World Health Organization. Guidelines for implementation article 13 and specific guidelines to address crossborder tobacco advertising, promotion and sponsorship and the depiction of tobacco in entertainment media for implementation of article 13 of the WHO FCTC. WHO; 2024. May 12, 2024. Accessed March 12, 2025. https://fctc.who.int/docs/librariesprovider12/meeting-reports/article-13-en-110424-ha.pdf?sfvrsn=2494287e_6&download=true

[10] Henriksen L. Comprehensive tobacco marketing restrictions: promotion, packaging, price and place. Tob Control. 2012;21(2):147-153. doi:10.1136/tobaccocontrol-2011-050416 22345238 PMC4256379

[11] European Union. Directive 2003/33/EC of the European Parliament and of the Council of 26 May 2003 on the approximation of the laws, regulations and administrative provisions of the Member States relating to the advertising and sponsorship of tobacco products. Official Journal of the European Union. June 20, 2003. Accessed March 12, https://eur-lex.europa.eu/legal-content/EN/TXT/PDF/?uri=CELEX:32003L0033

[12] European Union. Directive 2014/40/EU of the European Parliament and of the Council of 3 April 2014 on the approximation of the laws, regulations and administrative provisions of the Member States concerning the manufacture, presentation and sale of tobacco and related products and repealing Directive 2001/37/EC. Official Journal of the European Union. April 29, 2014. Accessed March 12, 2025. https://eur-lex.europa.eu/legal-content/EN/TXT/PDF/?uri=CELEX:32014L0040

[13] European Union. Directive 2010/13/EU of the European Parliament and of the Council of 10 March 2010 on the coordination of certain provisions laid down by law, regulation or administrative action in Member States concerning the provision of audiovisual media services (Audiovisual Media Services Directive). Official Journal of the European Union. April 15, 2010. Accessed March 12, 2025. https://eur-lex.europa.eu/legal-content/EN/TXT/PDF/?uri=CELEX:32010L0013

[14] Joint Action on Tobacco Control 2. Task 8.3a, TAPS report: Map and assess TAPS loopholes and best practices for TAPS bans. Joint Action on Tobacco Control 2. Accessed May 14, 2024. https://jaotc.eu/wp-content/uploads/2024/12/M8.2-Consultation-to-experts-on-TAPS.pdf

[15] European Union. Study on smoke-free environments and advertising of tobacco and related products. Specific contract 2019 71 01 implementing FWC No SANTE/2016/A1/39 - Lot1: Executive Summary. European Union; 2021. Accessed April 24, 2024. https://op.europa.eu/en/publication-detail/-/publication/66e35611-5d59-11ec-9c6c-01aa75ed71a1/language-en

[16] European Union. Study on smoke-free environments and advertising of tobacco and related products. Specific contract 2019 71 01 implementing FWC No SANTE/2016/A1/39 - Lot1: Study Appendices. European Union; 2021. Accessed April 24, 2024. https://op.europa.eu/en/publication-detail/-/publication/19bcb4c3-5d5a-11ec-9c6c-01aa75ed71a1/language-en

[17] European Union. Special Eurobarometer 506: Attitudes of Europeans towards tobacco and electronic cigarettes. European Union; 2021. Accessed April 24, 2024. https://op.europa.eu/en/publication-detail/-/publication/c070c04c-6788-11eb-aeb5-01aa75ed71a1

[18] Freeman B, Watts C, Astuti PAS. Global tobacco advertising, promotion and sponsorship regulation: what’s old, what’s new and where to next? Tob Control. 2022;31(2):216-221. doi:10.1136/tobaccocontrol-2021-056551 35241591

[19] WHO Framework Convention on Tobacco Control. Tobacco advertising, promotion and sponsorship: depiction of tobacco in entertainment media. Report by the Working Group. WHO Framework Convention on Tobacco Control. June 9, 2023. Accessed April 24, 2024. https://storage.googleapis.com/who-fctc-cop10-source/Main%20documents/fctc-cop10-8-en.pdf

[20] Getting Ahead of the Next Frontier in Tobacco Marketing. Vital Strategies. November 17, 2023. Accessed April 24, 2024. https://www.vitalstrategies.org/getting-ahead-of-the-next-frontier-in-tobacco-marketing/

[21] Leung J, Lim C, McClure-Thomas C, et al. Adolescent Exposure to Online Advertisements and Promotions for Tobacco Products on the Internet-A Cross-Sectional Analysis of the Global Youth Tobacco Surveys. J Adolesc Health. 2023;73(6):1138-1144. doi:10.1016/j.jadohealth.2023.07.019 37737754

[22] Kong G, Laestadius L, Vassey J, et al. Tobacco promotion restriction policies on social media. Tob Control. 2024;33(3):398-403. doi:10.1136/tc-2022-057348 36328589 PMC10154427

[23] Conference of the Parties to the WHO Framework Convention on Tobacco Control. Tobacco advertising, promotion and sponsorship: depiction of tobacco in entertainment media. WHO Framework Convention on Tobacco Control; 2016. Accessed April 24, 2024. https://fctc.who.int/docs/librariesprovider12/meeting-reports/fctc_cop_7_38_en.pdf?sfvrsn=7d942934_16&download=true

[24] Conference of the Parties to the WHO Framework Convention on Tobacco Control. Measures that would contribute to the elimination of cross-border advertising, promotion and sponsorship. WHO Framework Convention on Tobacco Control. February 16, 2009. Accessed April 24, 2024. https://apps.who.int/gb/fctc/PDF/cop3/FCTC_COP3_DIV3-en.pdf

[25] WHO Framework Convention for Tobacco Control. Protocol to eliminate illicit trade in tobacco products. World Health Organization. 2013. Accessed April 24, 2024. https://fctc.who.int/docs/librariesprovider12/default-document-library/protocol-to-eliminate-illicit-trade-in-tobacco-products.pdf?sfvrsn=a3a34d_1&download=true

[26] Peeters S, Gilmore AB. How online sales and promotion of snus contravenes current European Union legislation. Tob Control. 2013;22(4):266-273. doi:10.1136/tobaccocontrol-2011-050209 22267980

[27] Robertson L, Cameron C, McGee R, et al. Point-of-sale tobacco promotion and youth smoking: a meta-analysis. Tob Control. 2016;25(e2):e83-e89. doi:10.1136/tobaccocontrol-2015-052586 26728139

[28] Ford A, MacKintosh AM, Moodie C, Kuipers MAG, Hastings GB, Bauld L. Impact of a ban on the open display of tobacco products in retail outlets on never smoking youth in the UK: findings from a repeat cross-sectional survey before, during and after implementation. Tob Control. 2020;29(3):282-288. doi:10.1136/tobaccocontrol-2018-054831 31088915 PMC7231456

[29] Tobacco Control Laws. Tobacco Control Laws. Accessed April 24, 2024. https://www.tobaccocontrollaws.org/

[30] Reimold AE, Lee JGL, Ribisl KM. Tobacco company agreements with tobacco retailers for price discounts and prime placement of products and advertising: a scoping review. Tob Control. 2023;32(5):635-644. doi:10.1136/tobaccocontrol-2021-057026 35074932 PMC9359804

[31] World Health Organization. Tobacco plain packaging: global status 2021 update. WHO; 2022. Accessed April 24, 2024. https://iris.who.int/bitstream/handle/10665/356900/9789240051607-eng.pdf?sequence=1

[32] Moodie C, Angus K, Stead M. Consumer Response to Standardized Tobacco Packaging in the United Kingdom: A Synthesis of Evidence from Two Systematic Reviews. Risk Manag Healthc Policy. 2021;14:1465-1480. doi:10.2147/RMHP.S272259 33883953 PMC8053612

[33] World Health Organization. WHO report on the global tobacco epidemic, 2023: protect people from tobacco smoke. WHO; 2023. Accessed April 24, 2024. https://iris.who.int/bitstream/handle/10665/372043/9789240077164-eng.pdf?sequence=1

[34] Evans-Reeves KA, Hiscock R, Lauber K, Gilmore AB. Prospective longitudinal study of tobacco company adaptation to standardised packaging in the UK: identifying circumventions and closing loopholes. BMJ Open. 2019;9(9):e028506. doi:10.1136/bmjopen-2018-028506 PMC6773294 31551373

[35] Fooks G, Gilmore A, Collin J, Holden C, Lee K. The Limits of Corporate Social Responsibility: Techniques of Neutralization, Stakeholder Management and Political CSR. J Bus Ethics. 2013;112(2):283-299. doi:10.1007/s10551-012-1250-5 23997379 PMC3755635

[36] Klein JD, Thomas RK, Sutter EJ. History of childhood candy cigarette use is associated with tobacco smoking by adults. Prev Med. 2007;45(1):26-30. doi:10.1016/j.ypmed.2007.04.006 17532370

