# Supplementary material for: Recommendations for updating regulations on advertising, promotion and sponsorship of tobacco and nicotine products in the European Union
Source: Tob Prev Cessat. 2025 May 29;11:10.18332/tpc/204275. doi: 10.18332/tpc/204275 (PMC12118587; doi:10.18332/tpc/204275)
Supplement: Supplementary file 1 [file TPC-11-28-s1.pdf]

Supplementary Table 1: List of 24 different TAPS areas, grouped into six TAPS themes.

**A) Billboards, posters and other types of advertising outside the home:**
1- Advertising outside home (e.g. billboards, posters at bus-stop, advertising in sports stadia, advertising in taxis and advertising in public transport;
2- Cinema advertising (e.g. prior to the movie).
**B) Points of sale, sample, giveaways, promotional items and direct marketing:**
3- Free samples, free gifts and promotional items (i.e. distribution of free tobacco product samples, or free gifts supplied by tobacco manufacturers, or tobacco-branded promotional items, in the street, in the mail/post, at events, in restaurants/bars/discotheques and any other retail outlets);
4- Free trial of products;
5- Competition or prize draws linked to products;
6- Products visible on display in shops, supermarkets and other retail outlets (i.e. products can be seen by costumers and are not required to be hidden behind shutters or curtains, or are not required to be stocked out of sight under a counter);
7- Advertising at point of sale in shops, supermarkets and other retail outlets (posters inside shops, posters on shops windows, branding on display units or vending machines, branding on other shop furniture and fittings).
**C) Printed media:**
8- National or local print advertising for the general public (e.g. national or local newspapers, magazines);
9- International print advertising for the general public (e.g. national or local newspapers, magazines);
10- Print advertising in the trade press (e.g. magazines and newsletters for tobacco traders and retailers).
**D) TV and radio and product placement:**
11- National or local TV advertising;
12- International TV advertising;
13- National or local radio advertising;
14- International radio advertising;
15- Product placement (i.e. manufacturers paying for their products to be featured in films and television programmes, or brand names mentioned in the likes of radio broadcasts);
16- Use of products in films or television without explicit mention of the brand;
17- Crosses with sponsorship (e.g. branding on race car).
**E) Internet, social media and mobile applications:**
18- Online sales by specialist retailers;
19- Wider sales channels (e.g. e-commerce websites);
20- Non-retailer websites (e.g. search engines, news services), social media, appstore or apps downloaded from appstores for mobile devices.
**F) Sponsorship, corporate responsibility, corporate promotion and other public relations tactics, brand stretching and imitation products:**
21- Sponsorship (i.e. financial support for cultural, sporting and other events, or for organisations);
22- Corporate Social Responsibility actions by tobacco companies (i.e. donations, funding for research or scholarship, corporate entertaining, and any other activities carried out by companies under the heading of corporate social responsibility);

23- Brand stretching and imitation products (i.e. companies producing non-tobacco products under their brand name, such as clothing, and tobacco companies selling e-cigarettes using the same brand name as tobacco products and which resemble tobacco products);
24- Corporate promotion and other public relations tactics (e.g. Mission Winnow, Unsmoke Your World, Foundation for a Smoke-Free World).

Supplementary Table 2: Characteristics of invited and participating experts in the consultation.

Country	Response rate	No. of invited experts	Invited experts working as*	No. of experts that responded	Type of institution for experts that responded**
Austria	75%	4	policymaker and regulator<br>researcher<br>NGO<br>researcher	3	government<br>university<br>NGO
Belgium	33%	3	NGO<br>public authority<br>public authority	1	government
Bulgaria	25%	4	policymaker<br>policymaker<br>policymaker<br>other - health promotion consultant -psychologist	1	government
Czech Republic	67%	3	researcher<br>policymaker<br>policymaker	2	government<br>government
Cyprus	100%	1	policymaker	1	government
Denmark	0%	2	senior adviser<br>special consultant, attorney	0	
Estonia	33%	3	enforcement, expert (tobacco advertising)<br>specialist (promotion and sponsorship)<br>specialist (promotion and sponsorship	1	government
Finland	100%	3	ministerial adviser (policymaker)<br>senior specialist (researcher)<br>executive director (NGO)	3	government<br>government<br>NGO
France	0%	4	NGO<br>NGO<br>researcher<br>policymaker	0	
Germany	67%	3	regulator<br>schientific officer<br>expert	2	government<br>government
Greece	0%	1	researcher	0	
Hungary	0%	2	enforcement<br>regulation	0	

Ireland	50%	2	regional chief environmental health officer<br>policymaker	1	government
Italy	25%	4	director of prevention department<br>director tobacco control unit<br>director national centre on addiction and doping<br>president	1	public research center
Latvia	50%	2	senior expert (policymake/regulator)<br>senior expert (enforcement)	1	government<br>public hospital
Lithuania	50%	2	enforcement<br>NGO	1	government
Luxembourg	25%	4	tobacco control<br>expert addiction prevention & health promotion<br>pneumologue<br>director of cancer fondation	1	NGO
Malta	50%	2	public health resident specialist (regulator)<br>environmental health (regulator, enforcer)	1	government
Netherlands	75%	4	policy advisor tobaco control moh<br>professor in tobacco control research<br>policy advisor tobacco control, NGO<br>project leader enforcement tobacco control	3	government<br>government<br>NGO
Poland	100%	2	main specialist in tobacco control<br>researcher	2	government<br>university
Portugal	67%	3	enforcement<br>media regulator	2	government<br>other
Romania	0%	3	project manager<br>former president<br>president	0	

Slovenia	50%	4	policymaker<br>enforcement<br>NGO<br>NGO	2	government<br>government
Spain	75%	4	lawyer/policymarker<br>NGO<br>NGO<br>researcher	3	government<br>NGO<br>university
Sweden	67%	3	lawyer in charge of enforcing rules on marketing<br>secretary general, PR-expert from previous work<br>nog senior policy advisor	2	government<br>NGO
Norway	133%	3	regulator/enforcement<br>NGO<br>researcher	4	government<br>government<br>government<br>NGO
United Kingdom	0%	2	researcher<br>researcher	0	
TOTAL		**77**		**38**	

*policymaker, regulator, researcher, enforcement, NGO, others

**government, university, NGO, public hospital, public research centre

Supplementary Table 3: Extent of TAPS problems by TAPS area.

	High		Moderate		Low, none, don't know		Total
**A) Billboards, posters and other types of advertising outside the home**	n	%	n	%	n	%	N
1- Advertising outside the home (billboards, posters at bus-stops, advertising in sports stadia)	1	3%		0%	37	97%	38
2- Cinema advertising		0%		0%	38	100%	38
**B) Points of sale, sample, giveaways, promotional items and direct marketing**							
3- Free gifts		0%	2	5%	36	95%	38
4- Free trial of products		0%	2	5%	36	95%	38
5- Competitions and price draws		0%		0%	38	100%	38
6- Products displayed in shops, supermarkets and other retail outlets		0%	2	5%	36	95%	38
7- Advertising at point of sale in shops, supermarkets or other retail outlets	2	5%	1	3%	35	92%	38
**C) Printed media**							
8- National or local advert print		0%		0%	38	100%	38
9- International print in news papers		0%	2	5%	36	95%	38
10- Print advertising in the trade press		0%		0%	38	100%	38
**D) TV and radio and product placement**							
11- National or local TV advert		0%	7	18%	31	82%	38
12- International TV		0%		0%	38	100%	38
13- National radio		0%		0%	38	100%	38
14- International radio		0%		0%	38	100%	38
15- Product placement	1	3%	2	5%	35	92%	38
16- Use of products in films or TV without mention of the brand	2	5%	2	5%	34	89%	38
17- Crosses with sponsorship		0%	2	5%	36	95%	38
**E) Internet, social media and mobile applications**							
18- Online sales	1	3%	4	11%	33	87%	38
19- Wider sales channels		0%	3	8%	35	92%	38
20- Non-retailer websites	3	8%	5	13%	30	79%	38
**F) Sponsorship, corporate responsibility, corporate promotion and other public relations tactics, brand stretching and imitation products**							
21- Sponsorship		0%	4	11%	34	89%	38
22- Corporate social responsibility	1	3%	4	11%	33	87%	38
23- Brand stretching		0%	1	3%	37	97%	38
24- Corporate promotion creating confusion	1	3%	3	8%	34	89%	38
	12	1%	46	5%	854	94%	912

Supplementary Table 4: Current gaps in TAPS regulation in the EU.

**1- General gaps:**
Lack of updating the TAPS regulations; substantial gaps in regulation of new products; challenges defining and regulating sponsorship, especially indirect sponsorship activities [14, 15].
**2- Billboards, posters, other types of advertising outside the home:**
Advertising in public spaces, newspapers or magazines, railway stations, airports, inflight magazines and national or local TV advertising [14, 15, 17].
**3- Points of sale, sample, giveaways, promotional items and direct marketing:**
In the points of sales, there are products visible on display, promotional items, sales promotion and free trial of products [14, 15]. Duty free sales are often exceptions to TAPS bans [14]. Promotion campaigns under the batch of corporate social responsibilities [14, 15].
**4- Internet, social media and mobile applications:**
There are many products' depictions in entertainment media content, TV shows, films, online social networks or blogs, non-retailer websites and streaming services. Widespread online TAPS (direct or indirect), especially for new tobacco and nicotine products [14-17]. Widespread influencer marketing on social media, many posts looking like festivals, summer scenes [14, 17].
**5- Sponsorship, corporate responsibility, corporate promotion and other public relation tactics, brand stretching and imitation products:**
Sponsorship of events in countries outside the EU but broadcasted in the EU, direct or indirect TAPS at sports events, cultural events, also with free distribution of products [14, 15, 17].
**6- Monitoring and enforcement of TAPS bans**
Finally, the gaps related to monitoring and enforcement of TAPS bans are lack of financial and human resources [14, 15]; administrative burdens or delays in addressing violations, high litigation costs, dealing with internationally operating companies with their own legal departments [14, 15, 17]; difficulties in monitoring online content, social media [14, 17].

Supplementary Table 5: Proposed solutions to address current gaps in TAPS regulation in the EU.*

**1- General solutions**
Clear, comprehensive legislation closing the current loopholes, keeping pace with rapidly evolving products and TAPS methods [14, 15].
Comprehensive EU level regulations harmonising and strengthening existing laws [14, 17].
Regulations covering all emerging products and devices, as well as channels [14, 15].
Broader definition of smoking, including smoking behaviour [14, 15, 17].
Mandatory reporting of the tobacco industry's promotional expenditures [14, 17].
**2- Points of sale, sample, giveaways, promotional items and direct marketing:**
Display bans [14].
**3- Internet, social media and mobile applications:**
Bans on online sales [14].
Regulations covering social media advertising more clearly; specific and stricter provisions for social media [14, 15, 17].
Guidance for and cooperation with social media, regulation of influencer regarding TAPS [14].
**4- Sponsorship, corporate responsibility, corporate promotion and other public relation tactics, brand stretching and imitation products:**
Comprehensive ban on corporate social responsibility actions and corporate promotion [14]
Comprehensive ban on production and distribution of items such as sweets, snacks and toys or other products that resemble cigarettes or products, devices and accessories [14].
**5- Monitoring and enforcement**
Improved efficiency of monitoring and enforcement systems, adequate resource allocation (human, financial, technical), reduced administrative burdens, increased enforcement power and administrative decisions, sanctions [14, 15, 17].
Harmonized enforcement system [17].
EU level compliance tool [14, 15, 17].
Increased cooperation among the EU Member States and other international exchange of best practices, EU coordination [14, 15, 17].
Cooperation with other relevant stakeholders (civil society organisations, citizens, NGOs, audio-visual services regulators) [14, 17].
Notifying the industry and other relevant entities about the regulations [14].

© 2025 Koprivnikar H. et al.
